# Differential responses of salivary cortisol, amylase, and chromogranin A to academic stress

**DOI:** 10.1371/journal.pone.0256172

**Published:** 2021-08-12

**Authors:** Manita Tammayan, Nattinee Jantaratnotai, Praewpat Pachimsawat

**Affiliations:** 1 Faculty of Dentistry, Department of Advanced General Dentistry, Mahidol University, Bangkok, Thailand; 2 Faculty of Science, Department of Pharmacology, Mahidol University, Bangkok, Thailand; University of Lübeck: Universitat zu Lubeck, GERMANY

## Abstract

Salivary biomarkers have been widely used to help diagnose stress, anxiety, and/or depression. This study aimed to compare the responses of three commonly investigated salivary stress biomarkers that represent the hypothalamic-pituitary-adrenal activity (cortisol; sCort) and the sympathetic activity (alpha-amylase; sAA and chromogranin A; sCgA), using academic oral presentation as a model of stress. Twenty postgraduate dental students attended the seminar class as presenter and audience. The presenters’ performances were evaluated by the instructors suggesting more stress than the audience. The saliva was collected two times: before attending class and after an academic presentation (for presenters) or during the class (for audience). The pulse rates (PR) were also recorded. The results showed that the levels of all three biomarkers, as well as PR, were significantly higher in the presenter group compared with the audience group; however, the changes were most prominent with sCort and sAA (99.56 ± 12.76% for sCort, 93.48 ± 41.29% for sAA, 16.86 ± 6.42% for sCgA, and 15.06 ± 3.41% for PR). When compared between pre-post presentation, the levels of sCgA were not different, while those of sCort and sAA were significantly increased. These results suggest more sensitive reactivity to academic stress of sCort and sAA compared with sCgA and that the response of sCgA did not necessarily follow sAA pattern even though both are claimed to reflect the sympathetic activity. More studies are needed to elucidate the roles of sCgA in stress.

## Introduction

Stress is a great topic of research interest because it can impact the quality of life and can finally lead to increased morbidity and mortality [1,2]. Various methods have been used for stress evaluation, e.g. collecting questionnaires [3,4], physical examination by evaluating heart rate, electrocardiogram, heart rate variability, skin conductance, or sweat production [5–7], and evaluation of biological markers indicating the physiological response to stress [8,9]. The body’s response to stress involves two major systems which are the hypothalamic-pituitary-adrenal (HPA) axis and the sympathetic-adrenal-medullary (SAM) system [10–14].

Activation of the HPA axis finally results in cortisol production by the adrenal cortex. It has an important role in organizing the body’s response to stress by modulating the metabolism, the immune function, and the inflammatory response for optimal energy expenditure to fight stress [11]. Cortisol can be found in saliva, urine, sweat, and hair. The level of salivary cortisol (sCort) is directly proportional to the blood level and its level can reflect the activity of the HPA axis [15]. Among various biological stress markers, cortisol is the most studied and it is regarded as a gold standard marker of stress [11,16].

Activation of the SAM system results in increased production of adrenaline and noradrenaline which modulate metabolism and cardiovascular response to prepare the body to fight stress both physical and psychological [14]. The half-lives of catecholamines are short (only a few minutes) and sampling from different sites gives unreliable responses, making it an unsuitable marker of the sympathetic activity [17]. Moreover, unlike cortisol, catecholamines in saliva are not stable at room temperature over a long period of time making storage an impractical issue for field study [18]. However, there are other representatives of sympathetic activity which are easily detected in saliva such as alpha-amylase (sAA) and chromogranin A (sCgA) [10,19,20].

sAA is one of the major protein components in saliva with digestive function. Application of noradrenaline or beta-adrenergic agonist can stimulate sAA release supporting the use of sAA as an indicator of sympathetic activity [14]. A variety of studies on stress have consistently found increased levels of sAA in response to stress and it is also generally used as a biomarker of stress [20,21]. Compared with sCort, sAA generally responds to stress faster rising within 5 min after the onset of stress while sCort level increases in 20 min [22].

CgA is not as widely used as sAA as a marker of stress having just been discovered in 1965 (compared with 1831 for sAA) [23,24]. CgA is the major component of the proteins of granins family which is found in the neuroendocrine system. It is co-released with catecholamines from chromaffin cells in the adrenal medulla and sympathetic nerve endings upon sympathetic stimulation [25]. Plasma CgA has been found to be increased in neuroendocrine tumors, hypertension, renal failure, heart failure, etc [26]. In human, the presence of sCgA in submandibular glands was just confirmed in 2005 [27] but it was previously shown in animal that sCgA is released upon stimulation with noradrenaline [10,28–30]. Such information suggests that sCgA could be similar to sAA as a proxy of sympathetic activity.

A large number of studies have confirmed the use of sCort and sAA as markers of stress as reviewed elsewhere [9,11,14, 20,21]. For sCgA, there are also many studies on its increased level in response to various models of both psychological and physical stress [30–36]. All three constituents have generally been accepted as the surrogate of two key neuro-endocrine systems for biological stress assessment; however, there are only several studies with concurrent investigation of sCort, sAA, and sCgA levels [32,33,37,38]. Moreover, these studies reported conflicting responses between sCgA and sAA or sCort patterns. Clearly, more studies are needed to gain more understanding on the role of sCgA in stress and help elucidate the validity of sCgA as a SAM system marker.

The current study aimed to compare the responses of two established stress markers (sCort and sAA) with sCgA to academic stress in students. We hypothesized that sCgA response should have a similar pattern with that of sAA since both are claimed to represent the sympathetic activity. A graded individual oral presentation in a postgraduate dental seminar class was employed as a model of stress with control being the audience in the class. This study could contribute to the knowledge on these three biomarkers as well as to help choose the optimal marker and conditions to study stress.

## Materials and methods

### Subjects

Twenty-six healthy volunteers who were studying in a 3-year postgraduate dental program, Faculty of Dentistry, Mahidol University, showed interest in the experiment after an announcement in the faculty. The volunteers were informed about the study and gave written informed consent. The inclusion criteria included: (1) participants who are older than 18 years old, (2) no underlying diseases or pregnancy, (3) no medication, and (4) no habitual smoking and alcohol drinking. Exclusion criteria included participants with alcohol consumption during the past 12 hours, consumption of food or beverage other than plain water during the past hour, having oral ulceration that increases bleeding tendency while collecting saliva [8,39], and unwillingness to participate at any time during the experiment.

### Procedures

The study protocol was approved by Faculty of Dentistry/Faculty of Pharmacy, Mahidol University, Institutional Review Board COA. No. MU-DT/PY-IRB 2019/045.0907 and was registered at thaiclinicaltrials.org The data were collected between July 2019 and April 2020. The research was conducted according to the principles expressed in the Declaration of Helsinki.

An oral presentation on patient treatment planning is a requirement in the curriculum of the postgraduate dental student training. It was chosen as a naturalistic stressor. The student had to present in front of many instructors, and classmates. The instructors would comment and ask questions to evaluate and grade the presenter. If the presenter was considered not having enough knowledge, it could result in revising the presentation. The audience was other postgraduate dental students who attended the class presentation without being graded. The students took turns being a presenter or an audience. So one student took part in the experiment at least twice serving both as presenter and audience.

Before attending the class, volunteers were asked to complete a questionnaire which comprised demographic data, general health information, sleep duration the night before the testing day, and visual analog scale (VAS). VAS is an emotional rating scale from 0 to 10, with 0 indicating no stress and 10 indicating maximal stress. Saliva was then collected and pulse rate was measured at 8 AM as baseline (before class) and during the class break for the audience (10 AM) or right after presentation for the presenter. So the second collection time varied among the presenters depending on when they got to present, however, the entire process took place in the morning (8–12 AM) according to the seminar timetable. Each presentation took approximately 40–50 minutes. The participants pooled the saliva for 4 min and passively drool into a 2-mL tube. All samples were later stored at -80°C until analysis.

### Saliva analysis

The frozen saliva was thawed and centrifuged at 1500g for 15 min. The supernatant was retrieved for measuring sCort, sAA, and sCgA levels. For sCort measurement, a competitive enzyme immunoassay kit (Salimetrics, State College, PA, USA) was employed according to the manufacturer’s instruction. The sensitivity of the kit can measure between 0.007 to 3 μg/dL. The intra- and inter-assay coefficients of variation were 7% and 11%, respectively. For sAA measurement, a hand-held biosensor (Nipro, Osaka, Japan) was employed as previously described [40]. Briefly, an aliquot of 25 μL saliva was put onto the detector’s pad and inserted to be read in a machine. The biosensor can measure sAA levels up to 200 U/mL with 10.2% coefficient of variation [41]. For sCgA measurement, an enzyme-linked immunosorbent assay kit (MyBioSource, San Diego, CA, USA) was employed according to the manufacturer’s instructions. The kit can measure between 30–9000 ng/L sCgA with intra- and inter-assay coefficients of variation less than 15%.

### Statistical analysis

The collected data were analyzed using SPSS statistics program version 18.0 with the statistical significance level set at *p* < 0.05 (IBM, Armonk, NY, USA). All results shown are means and standard error of means (± SEM). Data were tested for normal distribution and homogeneity of variance using a Kolmogorov–Smirnov and Levene’s test before statistical procedures were applied. Two-sided paired t-test was used to compare the mean difference of salivary parameters in the same participant when he/she served as a presenter and an audience. Pearson’s correlation coefficient (*r*) was determined for the relationship between stress biomarker levels to VAS or sleep duration. The effect of different academic years in postgraduate students on stress biomarkers was assessed by Kruskal-Wallis test.

## Results

### Demographic data

A total of 20 students completed the experiment, six were excluded due to incomplete saliva collection. The participants’ age ranged from 26–33 years old. The mean age was 28.05 ± 1.70 years old. The body mass index (BMI) was between 16.75–26.5 kg/m^2^ and the average BMI was 20.52 ± 2.52 kg/m^2^. Because the number of male students was too small (n = 2), a separate analysis for each gender was not performed.

### Salivary cortisol

**Fig 1:**
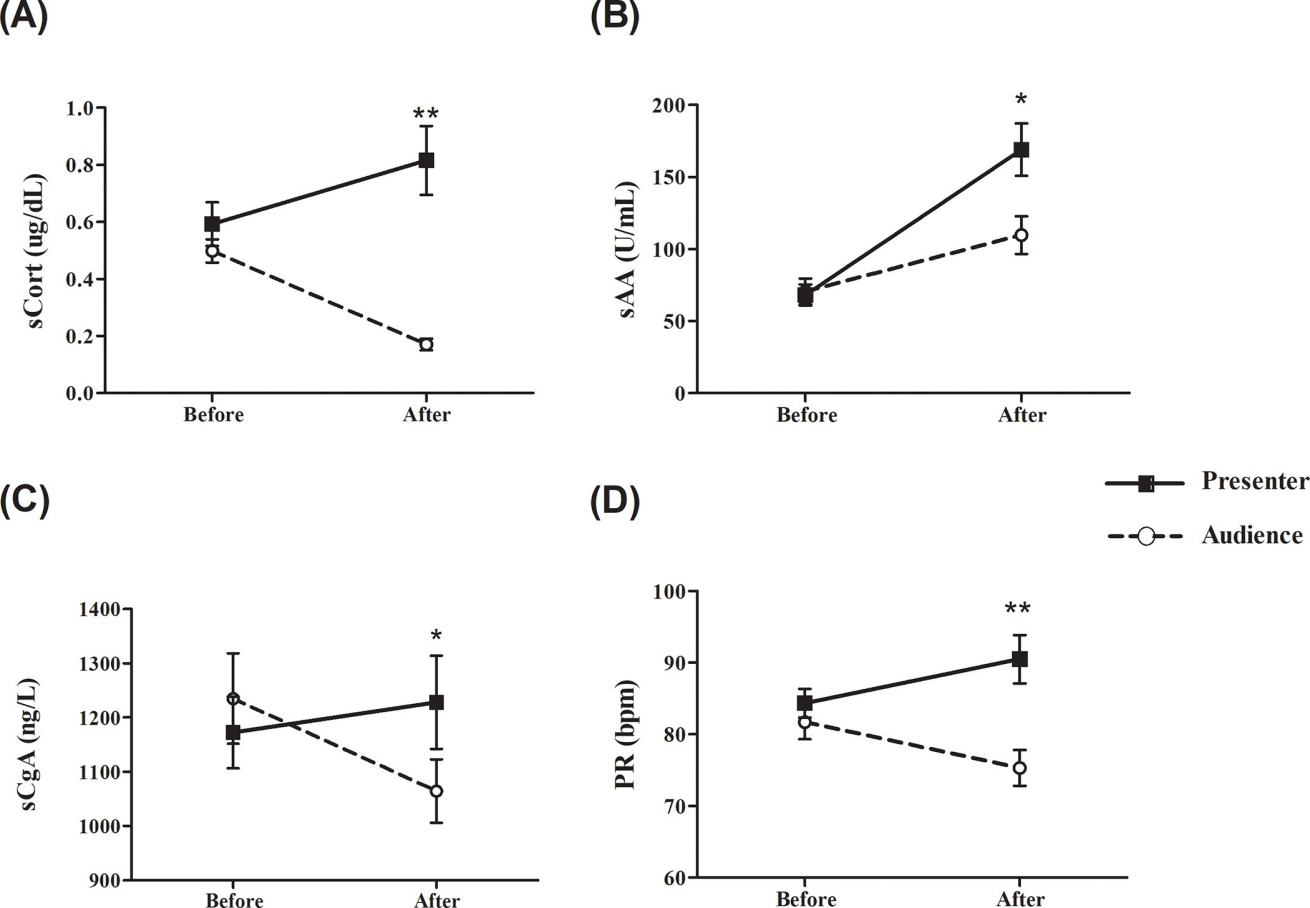
The levels of salivary biomarkers and pulse rate before class and after oral presentation (in presenters) or during class (in audience). Data were presented as mean ± SEM of salivary cortisol (A), salivary alpha-amylase (B), salivary chromogranin A (C) levels, and pulse rate (D). *p < 0.05 and **p < 0.001 compared with audience.

**Fig 2:**
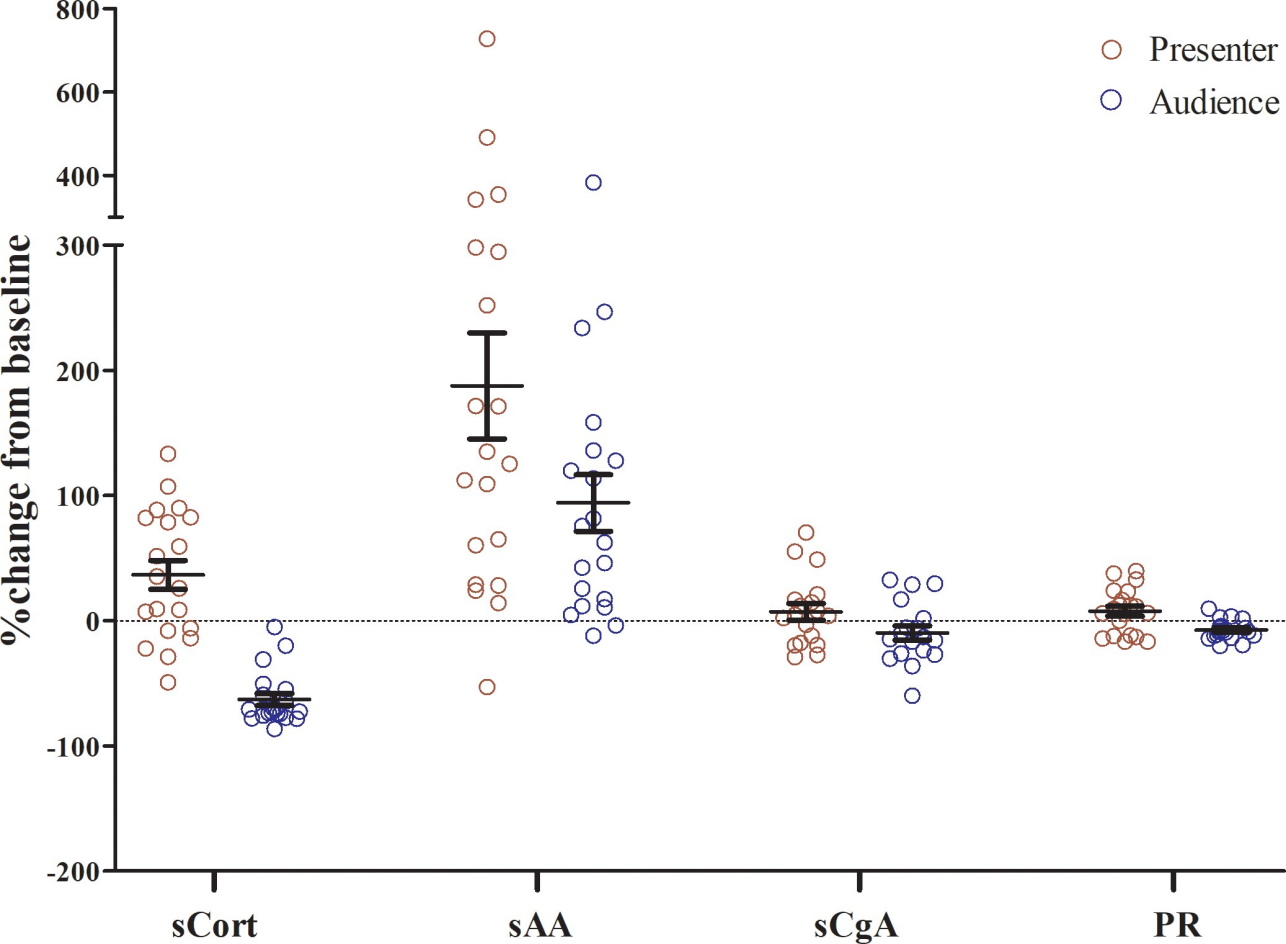
The percentage change of the salivary biomarkers and pulse rate from baseline. Data were presented as mean ± SEM.

The baseline sCort levels before class at 8 AM varied greatly between 0.227–1.355 ug/dL. The mean sCort concentrations (*n* = 20) were similar between the presenter and the audience groups (*p* = 0.250; Table 1). Then, sCort changes between two groups showed the opposite trend. After the class began, sCort levels in the audience decreased while those of the presenters’ increased significantly after their presentation (Fig 1A). The difference in sCort levels after class between the audience and the presenter groups were also significant (*p* < 0.001). The percentage of sCort change from baseline in the presenter group was 36.65 ± 11.53% while the change in the audience group was -62.91 ± 4.76% as shown in Fig 2. The percentage difference between presenter and audience was 99.56 ± 12.76% (*p* < 0.001).

**Table 1:** Mean salivary cortisol, amylase, chromogranin A levels, and pulse rates before class and after oral presentation (in presenters) or during class (in audience).

Parameters	group	Before	*p*	After	*p*	Before—after	*p within*	*p between*
		mean±SEM		mean±SEM		mean±SEM	*group*	*group*
**sCort**	Presenter	0.593±0.076	**0.250**	0.816±0.121	< 0.001*	0.223±0.075	0.008*	< 0.001*
(ug/dL)	Audience	0.499±0.041		0.171±0.021		-0.328±0.043	< 0.001*	
**sAA**	Presenter	68.15±7.26	**0.809**	169.05±18.17	0.026*	100.90±17.27	< 0.001*	0.007*
(U/mL)	Audience	70.63±8.95		109.68±13.01		39.05±10.42	0.001*	
**sCgA**	Presenter	1172.57±66.14	**0.459**	1227.99±85.77	0.034*	55.42±76.30	**0.478**	0.012*
(ng/L)	Audience	1234.96±83.43		1064.58±58.54		-170.37±91.76	**0.081**	
**PR**	Presenter	84.35±2.01	**0.344**	90.50±3.39	< 0.001*	6.15±3.29	**0.077**	< 0.001*
(bpm)	Audience	81.72±2.41		75.31±2.50		-6.41±1.49	< 0.001*	

### Salivary alpha-amylase

The levels of sAA at baseline before class in the presenter and the audience groups were similar as shown in Fig 1B and Table 1 with a wide range of sAA activity between 16–134 U/mL. In contrast to the sCort pattern, the levels of sAA in both the presenter and the audience groups increased after class with the increase in the presenter groups significantly more than that in the audience group (2.48 vs 1.55 fold increase, respectively). The percentage difference of sAA levels from baseline in the presenters was 187.64 ± 42.25% compared with 94.17 ± 22.57% in the audience (Fig 2). The percentage difference between presenter and audience was 93.48 ± 41.29% (*p* < 0.05).

### Salivary chromogranin A

The participants showed a wide range of sCgA levels between 653–3264 ng/L. At baseline, there was no difference in sCgA levels between the presenter and the audience groups (*p* = 0.459) as shown in Fig 1C (*n* = 18). The patterns of sCgA changes after oral presentation and during class were similar to those of the sCort changes with the levels of sCgA increasing in the presenters and decreasing in the audience. However, the reactivity of sCgA was much less than those of sCort and sAA as the percentage changes from baseline of sCgA were only 7.22 ± 6.64% in the presenters and -9.64 ± 5.78% in the audience (Fig 2). Still, these numbers represented a significant difference between the presenter and the audience groups (*p* = 0.018). The percentage difference between presenter and audience was 16.86 ± 6.42% (*p* < 0.05).

### Pulse rate

No significant differences were seen in pulse rates prior to class attendance between the presenter and the audience groups (*p* = 0.344; *n* = 20). The pattern of pulse rate fluctuation after class on both groups was consistent with those of sCort and sCgA patterns showing an increase in the presenters and a decrease in the audience as shown in Fig 1D. This resulted in a significant difference (*p* < 0.001) in pulse rates between the presenter (7.70 ± 4.02%) and the audience (-7.35 ± 1.68%) as shown in Fig 2. The percentage difference between presenter and audience was 15.06 ± 3.41% (*p* < 0.001).

### Relationship between salivary biomarkers and other variables

**Fig 3:**
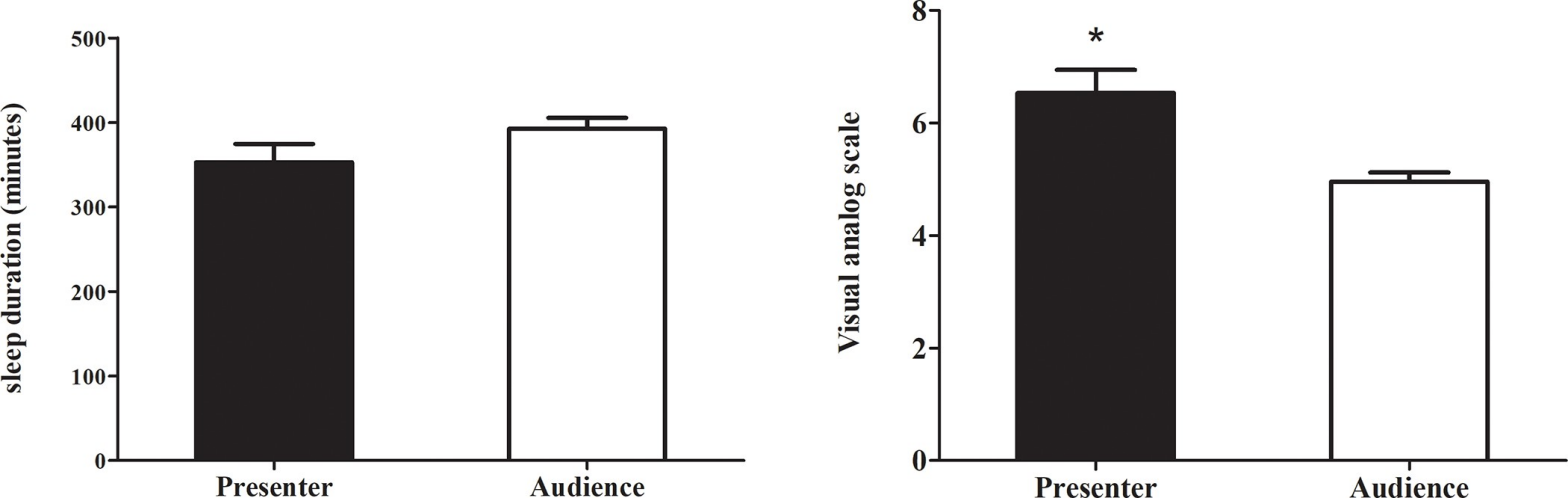
VAS scores and sleep duration between presenters and audience. Data were presented as mean ± SEM. *p < 0.001 compared with audience.

The mean VAS scores in the presenters (6.53 ± 0.41) and the audience (4.95 ± 0.17) were significantly different (*p* < 0.001; Fig 3). Correlation analyses revealed that the larger the difference in sAA changes in the presenters, the higher the VAS scores (*r* = 0.569; *p* = 0.027). The mean sleep duration the night before class was 5:45 h in the presenters, and 6:31 h in the audience which were not significantly different (*p* = 0.071; Fig 3). Examining the relationship between VAS scores and sleep duration to sCort, sAA, sCgA, and pulse rate responses revealed no significant correlations in the presenter group (S1 Fig). We hypothesized that students in the higher academic year might show less stress compared to first-year students who had to give oral presentation for the first time; however, there was no difference in the biomarker levels among students of different academic years.

## Discussion

This study employed a naturalistic academic situation where students had to integrate the knowledge to present the treatment planning of their patients as well as answering comments and questions in a room full of instructors and classmates. The results found the levels of all three biomarkers examined were increased in the presenter group compared with the audience group. These results indicated that this was indeed a stressful situation that caused stress to the presenters but not to the audience. This situation can be an appropriate model for studying psychosocial stress in the presenters where the audience serves as control.

Some previous studies chose to collect saliva on a different day from the stressful day (exam day or oral presentation day) due to the assumption that the baseline levels of the biomarkers might be higher from the presence of stress even before the stressful event occurred [42,43]. The present study revealed that investigating the biomarker levels on the same day can be performed. There was no difference in baseline levels of sCort, sAA, and sCgA between the presenter and the audience groups before class suggesting the acute nature of stress. Interestingly, the anticipation of stress did not significantly affect the objective stress response (biomarker levels) while subjective stress response (VAS) was significantly higher in the presenters.

The patterns of the biomarker change in the audience (a control group) were different, i.e. a significant decrease from baseline in sCort level, an increase in sAA level, and no change (nonsignificant decrease; p = 0.081) in sCgA level. One reason behind these different patterns could lie in the baseline diurnal variation especially in the morning when the experiment was performed. sCort showed a typical diurnal rhythm of a peak within 30 min after awakening then a sharp decrease in the morning period followed by a much flatter slope in the afternoon reaching a nadir in the evening [44–46]. The sCort change from baseline in the audience matched its downward diurnal pattern in the morning. For sAA, the trend was the opposite with a drop soon after awakening followed by a sharp increase in the morning period then it somewhat stabilized into the evening [44]. Hence, the increase in sAA level in the audience as seen in the current study. The diurnal pattern of sCgA was reported in only one previous study which showed a high level upon awakening followed by a sharp decrease an hour later [45]. Then it remained low throughout the day until an increase in the evening displaying a U-shape pattern. This one was elusive to interpret as sCgA level was not significantly decreased in the audience, it could be considered a match with its diurnal pattern of stable level in the morning. More studies are needed to confirm the diurnal pattern of sCgA as this was the only study we could find to compare with our results.

The increases in sCort and sAA levels from baseline in the presenter group were significant (36.65% and 187.64%, respectively) suggesting that stress was much more pronounced than their diurnal patterns. Additionally, changes in sCort and sAA activities were different compared with the audience group that received no stress (99.56% and 93.48%, respectively). These findings were in agreement with those reported in the studies examining academic performance situations [42,43,47–50]. Interestingly, the values compared with baseline levels of sAA (187.64%) appeared more remarkable than that of sCort (36.65%) but when compared with control they were in the same range at about 90%. This could be because of different baseline diurnal variations which showed a downward trend for sCort and an upward trend for sAA. Normally, sCort showed a downward trend in the morning, so stress didn’t appear to have that much effect on sCort increase compared with baseline before presentation. While sAA normally showed upward trend so the levels appeared to increase remarkably with stress. Still, these numbers suggested that stress override the baseline diurnal pattern. Even though it might be suggested that performing the experiment in the afternoon would evade the interference from the fluctuation of the diurnal pattern better, it does not seem significant in case of strong level of stress. However, in case of mild stress, the detection of changes in these biomarkers might not be as obvious thus it is suggested that if possible performing the experiment in the afternoon seems to be a safer option.

Even though the sCgA levels between the presenter and the audience groups were significantly different (16.86%), the levels in the presenter group were not significantly different pre and post presentation (7.22%) suggesting a mild reactivity to stress compared with sCort and sAA. Filaire et al also found increased levels of sCort and sAA but not sCgA in 52 professors giving lectures to a large audience compared with non-teaching day [33]. Similarly, Chennaoui et al. also found no change in sCgA levels among 9 high-level swimmers during competition [32]. The levels of sCort and sAA also increased pre and post swimming but they were increased at different types of competition. In contrast, two studies measuring sAA and sCgA levels found both to increase after high-intensity physical exertion [36,51]. There are still not many studies comparing the responses of these biomarkers especially sAA and sCgA, both of which are claimed to represent SAM system activity. These conflicting results could lie in many factors such as the model of stress (psychological or physical) or timing of saliva collection. Moreover, sCgA is released from only the submandibular glands, not all salivary glands [27], the collection methods may affect its concentration in saliva.

We hypothesized that sleep duration could affect the levels of the salivary biomarkers and it was found that sleep deprivation and poor sleep quality could impair mood, memory, and academic performances triggering stress in students [52,53]. Previous studies have reported the effect of poor sleep and blunted sCort reactivity and a negative correlation between sleep duration and sCort and sAA levels [32,54]. However, no correlation was observed between sleep duration and any of the salivary biomarker responses. It could be that in the current study the nature of stress was acute and the duration of sleep between the presenters and the audience was not significantly different.

These salivary biomarkers could be useful indicators for stress evaluation since measuring their levels provides an objective response of the body which is more reliable than using a questionnaire or other subjective stress assessment. We have found a portable device that can measure sAA levels on-site to be reliable and consistent [40,55,56]. However, its limitation lies in the inability to process a large number of the samples in a short period. It might not be practical in case of a large sample size since timing plays an important role in determining stress at a particular time point. So the application of using saliva to monitor stress real-time and on-site is still in its early stage of practice. More recently, the wearable biosensor that can measure cortisol in sweat is being developed [57,58]. This could be very important in stress research to measure cortisol real-time and it can be applied to be used like a smart watch to monitor stress in the future.

There are a few limitations in this study. First, the participants were predominantly female (90%) so it might not represent the real population. Even though the participants using contraceptives or hormonal drugs were excluded, the menstrual cycle was not controlled. However, previous studies did not find gender to affect sAA and sCort responses to academic stress [47,50]. Second, we did not measure the levels of the biomarkers continuously or with more time points so it was impossible to establish the timeline of these biomarkers but generally sAA and sCgA responded faster to stress while sCort showed a delayed response [38]. In our study, the presentation lasted about 40–50 min which was long enough for all biomarkers to show elevated response. Another limitation was using subjective stress assessment at only one time point. The participants scored their stress levels using VAS at baseline (8 AM) but not after class, making it difficult to examine the anticipatory relationship between subjective stress and the biomarker activities. Additionally, proper self-report instrument which is more specific to academic stress such as State-Trait Anxiety Inventory [43,48,50] and academic stress inventory questionnaire [49] may reflect the stress better.

## Conclusion

The present study compared sCort, sAA and sCgA responses to academic stress. All three biomarker activities in the stress group were increased compared with the control group. However, when compared within the presenter group, only sCort and sAA levels showed significant elevation after presentation. Our results indicated that sCort and sAA were more sensitive and reliable biomarkers than sCgA in academic stress. Much is still unknown about the nature of response of sCgA to stress and more studies comparing sAA and sCgA at various time points are needed.

## Supporting information

S1 Fig The correlation between the biomarkers and pulse rate with VAS and sleep duration. (TIF)
