# Supplementary figures and images for: Differential responses of salivary cortisol, amylase, and chromogranin A to academic stress
Source: PLoS One. 2021 Aug 12;16(8):e0256172. doi: 10.1371/journal.pone.0256172 (PMC8360508; doi:10.1371/journal.pone.0256172)

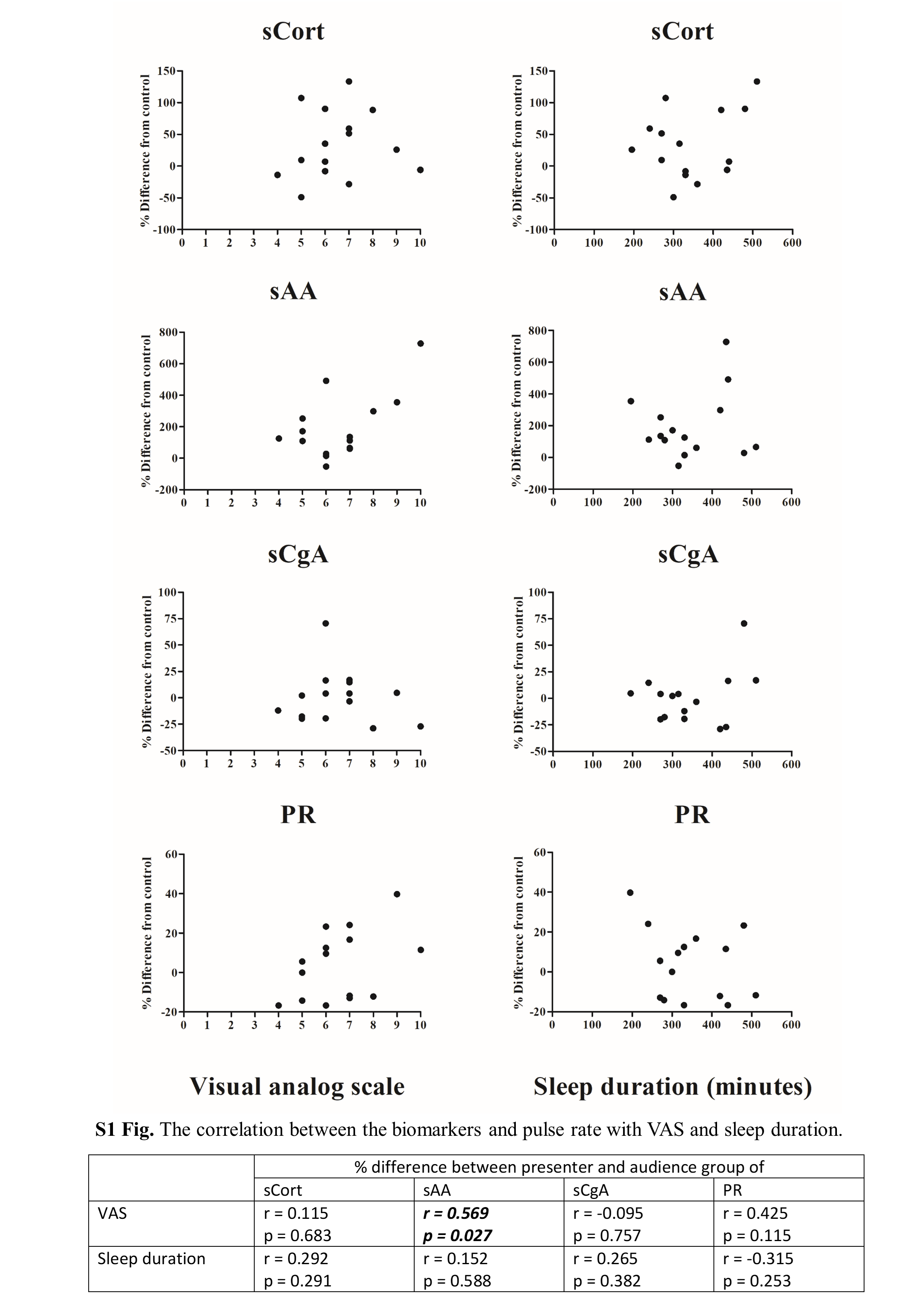

Supplement: S1 Fig — (TIF) [file pone.0256172.s001.tif]
